# Systematic review update of observational studies further supports aspirin role in cancer treatment: Time to share evidence and decision-making with patients?

**DOI:** 10.1371/journal.pone.0203957

**Published:** 2018-09-25

**Authors:** Peter C. Elwood, Janet E. Pickering, Gareth Morgan, Julieta Galante, Alison L. Weightman, Delyth Morris, Marcus Longley, Malcolm Mason, Richard Adams, Sunil Dolwani, John Chia W. K., Angel Lanas

**Affiliations:** 1 Cochrane Institute of Primary Care and Public Health, Cardiff University, Cardiff, United Kingdom; 2 Institute of Food, Nutrition and Health, University of Reading, Reading, United Kingdom; 3 Hywel Dda University Health Board, Llanelli, United Kingdom; 4 Department of Psychiatry, University of Cambridge, Cambridge, United Kingdom; 5 Specialist Unit for Review Evidence, Cardiff University, Cardiff, United Kingdom; 6 Health Policy, University of South Wales, Pontypridd, United Kingdom; 7 Division of Cancer and Genetics, School of Medicine, Cardiff University, Cardiff, United Kingdom; 8 Institute of Cancer & Genetics Cardiff University, Cardiff, United Kingdom; 9 Division of Population Medicine, School of Medicine, Cardiff University, Cardiff, United Kingdom; 10 Division of Medical Oncology, National Cancer Centre, Singapore, Singapore; 11 University of Zaragoza, IIS Aragón, CIBERehd, Zaragoza, Spain; Southern Illinois University School of Medicine, UNITED STATES

## Abstract

**Background:**

Evidence is growing that low-dose aspirin used as an adjuvant treatment of cancer is associated with an increased survival and a reduction in metastatic spread. We therefore extended up to August 2017 an earlier systematic search and meta-analyses of published studies of low-dose aspirin taken by patients with a diagnosis of cancer.

**Methods:**

Searches were completed in Medline and Embase to August 2017 using a pre-defined search strategy to identify reports of relevant studies. References in all the selected papers were scanned. Two reviewers independently applied pre-determined eligibility criteria and extracted data on cause-specific cancer deaths, overall mortality and the occurrence of metastatic spread. Meta-analyses were then conducted for different cancers and heterogeneity and publication bias assessed. Sensitivity analyses and attempts to reduce heterogeneity were conducted.

**Results:**

Analyses of 29 studies reported since an earlier review up to April 2015 are presented in this report, and these are then pooled with the 42 studies in our earlier publication. Overall meta-analyses of the 71 studies are presented, based on a total of over 120 thousand patients taking aspirin. Ten of the studies also give evidence on the incidence of metastatic cancer spread.

There are now twenty-nine observational studies describing colorectal cancer (CRC) and post-diagnostic aspirin. Pooling the estimates of reduction by aspirin which are reported as hazard ratios (HR), gives an overall HR for aspirin and CRC mortality 0.72 (95% CI 0.64–0.80). Fourteen observational studies have reported on aspirin and breast cancer mortality and pooling those that report the association with aspirin as a hazard ratio gives HR 0.69 (0.53–0.90). Sixteen studies report on aspirin and prostate cancer mortality and a pooled estimate yields an HR of 0.87 (95% CI 0.73–1.05). Data from 12 reports relating to other cancers are also listed. Ten studies give evidence of a reduction in metastatic spread; four give a pooled HR 0.31 (95% CI 0.18, 0.54) and five studies which reported odds ratio of metastatic spread give OR 0.79 (0.66 to 0.95).

**Conclusion:**

Being almost entirely from observational studies, the evidence of benefit from aspirin is limited. There is heterogeneity between studies and the results are subject to important biases, only some of which can be identified. Nevertheless, the evidence would seem to merit wide discussion regarding whether or not it is adequate to justify the recommendation of low-dose therapeutic aspirin, and if it is, for which cancers?

## Introduction

The role of low-dose aspirin prophylaxis is well established in vascular disease [1,2] and in the reduction of colorectal, and probably other cancers [3–5], and it has been predicted that that ‘prevention of cancer could become the main justification for aspirin use’ [6].

The earliest reports on aspirin and cancer described a reduction in metastatic spread and focused on the role of platelets, consistent with a treatment, rather than a preventive effect [7]. Later, evidence of effects of aspirin on certain biological mechanisms relevant to cancer growth and to metastatic spread [8–10] justified an expectation of benefit from aspirin treatment of cancer. Some of the long-term follow-up studies of early vascular trials gave evidence of reductions attributable to aspirin in the metastatic spread of a range of cancers in subjects who had been free of metastases at diagnosis [11] again suggesting a treatment effect of aspirin. Furthermore, while there is usually a delay before evidence of a reduction in incident cancer becomes apparent, a reduction in mortality appears to commence without any delay in patients who already have metastases, again suggesting a treatment effect of aspirin [6].

On the other hand, the treatment of cancer by aspirin has been examined in only a very few *ad hoc* randomised controlled trials, and these were either unrealistically small [12–14], or randomisation was compromised [15]. The bulk of clinical evidence on aspirin taken by patients with cancer is therefore limited to observational studies.

Concurrently, patients with cancer are being recruited into randomised trials of aspirin as an adjunct treatment [16,17]. It will however be perhaps 10–15 years before evidence from these begins to become available, and it is likely that trials will focus on only a very few of the most common cancers. In the meantime, evidence from observational studies is, by default, of considerable and immediate importance.

In 2016 we reported a systematic review and meta-analysis of all the reports then available on aspirin taken by patients with cancer [18] Since then new evidence has been reported which we believe justifies a re-evaluation of the topic. In what follows we therefore summarise the new evidence and we follow this with meta-analyses of all the available relevant studies. In particular, we summarise the evidence on the three main cancers: colorectal, breast and prostate, and we then list the evidence from single studies of other cancers. All this leads to a presentation of what we believe is a basic issue: whether or not the present evidence is adequate to justify the informing of patients with cancer, and if it is adequate, then patients with which cancers?

## Methods

In 2015 we reported the results of a systematic review and meta-analysis of all available reports published up to March 2015 [18]. We have now updated that search to August 2017 and we present a review and meta-analysis of all the recent papers identified in a search from April 2015 to August 2017 inclusive. We then present a summary, meta-analyses and interpretation of all the available reports on the topic.

The procedures adopted throughout followed the PRISMA guidelines [19] and a full description of the search strategy is given in S1 File. In brief: systematic searches using key words were conducted in Medline and Embase, limited to human studies in peer-reviewed journals. Studies were selected by two authors (PE and GM) if (a) the studied population comprised patients diagnosed with cancer; (b) aspirin was taken regularly after cancer diagnosis; (c) the studies were randomised trials, case-control studies or cohort studies. Data on cancer specific and all-cause mortality, and data on the incidence of metastatic spread and adverse effects attributable to aspirin were extracted. Reference lists of the relevant studies identified were searched for relevant reports. Many of the authors were written to for additional details and authors of all the studies were asked specifically about gastrointestinal bleeding in the patients included in their study.

The methodological quality of the studies was assessed and graded independently by two authors (AW and PE) using the Newcastle-Ottawa Scale [20] (see S2 File). Disagreements in grade on a nine point scale, were discussed and agreed.

Meta-analyses were conducted, using a 'random effects' model. Analyses were performed first on the new papers published since April 2015, and then on all the available studies from both the earlier search and the new search were pooled and meta-analyses performed. Egger's test [21] was used in looking for publication bias and funnel plots were created to highlight outlying studies. Results of attempts to reduce heterogeneity were based on the omission of studies which, on the tree diagrams and sensitivity analyses identified studies which were outlying or appeared to have an excessive influence on the pooled hazard ratios. The detailed work on all this is given in S3 File.

## Results

A literature search (April 2015 to August 2017) to up-date our earlier report [18] identified 229 new reports additional to the 640 papers we had already identified up to March 2015 [18]. Thirty-one of the 229 new reports were judged to be of relevance and on inspection of the full text 29 fulfilled the criteria for inclusion. A summary of each of these, together with an estimate of quality according to the Newcastle-Ottawa assessment protocol (see S2 File).

No new randomised trial was identified in the up-dating search, but there were 13 new reports of observational studies of aspirin and colorectal cancer, four new reports in patients with breast cancer, six of patients with prostate cancer and six of patients with other cancers. Six reports gave evidence on metastatic spread. Details of these, including the published estimates of both the cause-specific and all-cause mortality are displayed in Table 1.

**Table 1:** Aspirin treatment of cancer in observational studies reported 2015–2017. For details of studies published before 2015, see our earlier report [18].

Study	Aspirin/no Aspirin	Events aspirin/none	Follow-up duration	Outcome	HR/RR (95% CI)	Comment
**COLORECTAL and GASTROINTESTINAL CANCER**						
Bains et al. [22]	6,102/17,06 0	1,158/5,375	3 years	Cause specific mortality	HR 0.85 (0.79, 0.92)	
				All-cause mortality	HR 0.95 (0.90, 1.01)	
Frows et al. [23]	1,008/8,278	5,138	Up to 15 years	Cause specific mortality	HR 0.44 (0.33, 0.58)	Time dependent survival analyses
				All-cause mortality	HR 0.52 (0.44, 0.63)	
Giamperie et al, [24]	20/46	8/43	6 years	Progression free survival	HR 0.48 (0.30, 0.79)	
				All-cause mortality	HR 0.43 (0.26, 0.72)	
Shimoike et al. [25]	148/343	**?**	Over 5 years	Cause specific mortality	HR 1.38 (0.84, 2.26)	Poster presentation. Our estimates of HR. Other antiplatelet drugs used
				All-cause mortality	HR 0.61 (0.28, 1.33)	
Restivo et al. [26]	37/204	?	37months (19-57m)	Prog free survival	HR 0.20 (0.07, 0.60)	
				Overrall survival	HR 0.21 (0.05, 0.89)	
Ventura et al. [27]	9,938/ 217,070	45/742	6 years	Cause specific mortality	HR 0.71 (0.52, 0.97)	‘No certainty that aspirin taking continued to death’
				All-cause mortality	HR 1.18 (1.12, 1.23)	
Gray et al. [28]	146/534	40/172	?	Cause specific mortality	HR 0.69 (0.47, 0.98)	PIK3CA and PTGS2 evaluated
				All-cause mortality	HR 0.76 (0.57, 1.03)	
Hua et al. [29]	676/1,397	17/61	11 years	Cause specific mortality	HR 0.44 (0.25, 0.71)	
				All-cause mortality	HR 0.75 (0.59, 0.95)	
Vietonmaki et al. [30]	676/1,397	413	15 years	Cause specific mortality	HR 1.28 (0.40, 4.12)	Competing risk analyses
Murphy et al. [31]	95/296	8/43	110 months	Cause specific mortality	RR 0.72 (0.34–1.53)	Data for mutant and wild PIK3CA combined
				All-cause mortality	RR 2.36 (1.44–3.87)	
Ratnsinghe et al. [32]	5,935/3,934	44/42 Males	17–21 years	M cause specific mortality	RR 0.68 (0.37. 1.26)	
	8.903/4,062	71/36 Females		F cause specific mortality	RR 1.61 (0.91, 2.85)	
Hippisley-Cox et al. [33]	4,528/39,617	?	1–25 years	Cause specific mortality	HR 0.81 (0.73, 0.90)	Male and female data combined
				All-cause mortality	HR 0.85 (0.78, 0.93)	
Hamada et al. [34]	269/348	37/81	11.5 years	Cause specific mortality	HR 0.65 (0.40, 1.07)	
Colorectal cancer deaths: **Pooled HR for eleven studies: 0.68** (0.57, 0.81), heterogeneity p<0.0005, Egger's test for bias p = 0.09 All cause deaths: **Pooled HR for nine studies: 0.76** (0.63–0.91) heterogeneity p<0.0005, Egger's test p = 0.04						
**BREAST CANCER**						
McMenamin et al. [35]	2,822/12,318	261/929	3–6 years	Cause specific mortality	HR 0.92 (0.75, 1.14)	
				All-cause mortality	HR 1.21 (1.04, 1.40)	
Shiao et al. [36]	65/157	11/50	Up to 10 years	Cause specific mortality	HR 0.41 (0.20, 0.83)	‘aspirin’ includes other anticoagulants
				All-cause mortality	HR 0.67 (0.35, 1.27)	
Ratnasinghe et al. [32]	8,903/4,062	84/47	17–21 years	Cause specific mortality	RR 0.82 (0.49, 1.36)	Two cohorts pooled
MsCarthy et al. [37]	60/52	n.a.	n.a.	Breast cancer recurrence	HR 0.65 (0.46, 0.91)	Aspirin and NSAID use. Includes data on PIK3CA
Breast cancer deaths: **Pooled HR for three studies: 0.70** (0.47–1.03) heterogeneity p = 0.04, Egger's test P<0.16 All cause deaths: **Pooled HR for two studies 0.98** (0.56–1.71) heterogeneity p = 0.08, Egger's test not possible						
**PROSTATE CANCER**						
Osborrn et al. [38]	147/142	2/5	6 years	Cause specific mortality	HR 0.20 (0.04, 1.13)	
Veitonmaki et al. [39]	332/6,205	23/592	7.5 years	Cause specific mortality	HR 0.62 (0.30, 1.32)	Estimates with ‘lag time’ ignored
Zhou et al. [40]	?/?	103/67	2–7 years	Cause specific mortality	HR 0.83 (0.72, 0.95)	Results of daily aspirin in two cohorts pooled
				All-cause mortality	HR 0.75 (0.66, 0.86)	
Cardwell et al. [41]	1,184/3,531	616/568	4–12 years	Cause specific mortality	OR 1.02 (0.78, 1.34)	
				All-cause mortality	OR 1.22 (1.02,1.45)	
Ratnasing et al. [32]	14,943/8,806	2,735/3,170	17–21 years	Cause specific mortality	RR 1.11 (0.60 2.05)	
Downer et al. [42]	3,277	190/307	n.a.	Cause specific mortality	HR 0.68 (0.52, 0.90)	Long-term follow-up of a previously randomised trial
				All-cause mortality	HR 0.72 (0.61, 0.84)	
**OTHER CANCERS**						
Bar et al. [43]	31/11	29/34	4 years	Recurrent-free survival	HR 0.52 (0.30, 0.90)	
	Ovarian		3–173 month	Overall survival	HR 0.50 (0.29, 0.84)	
Matsuo et al. [44]	158/1,529	127	31 months	Disease specific	HR 0.46 (0.25, 0.86)	
	Endometrium.			Overall survival	HR 0.23 (0.08, 0.64)	
Li et al. [45]	60/60 Liver	?	80 months	Total mortality	HR 0.60 (0.35, 1.03)	Matched pairs
Veitonmaki et al. [30]	7,183/17,509	19/6 6/4	15 years	Disease specific	HR 1.27 (0.57, 2.83)	
	Lung Pancreas			Disease specific	HR 0.85 (0.24, 3.05)	
Maddison et al. [46]	60/60	284 total	?5 years	Disease specific	HR 1.00 (0.73,1.37)	
	Lung					
Kim et al. [47]	Head & neck	81/1311	24–192 months	Disease specific	HR 1.30 (0.78, 2.18)	
				Overall survival	HR 1.30 (0.96, 1.92)	

These results confirm the earlier findings we reported [18]. For colorectal cancer our earlier review of 16 observational studies had given a pooled HR 0.71 (95% CI 0.58, 0.87) and this more recent review gives HR = 0.68 (0.57, 0.81). Our earlier report gave a pooled estimate based on 10 studies of breast cancer as HR = 0.68 (95% CI 0.46, 1.02), and the estimate based on the recent reports is HR 0.70 (0.47–1.03). For prostate cancer the two estimates are not as close, the earlier series of ten reports giving HR 0.94 (95% CI 0.76, 1.17) and the six new studies, when pooled, yield an HR of 0.74 (0.59–0.92).

These pooled results for the studies published up to March 2015 and studies published since April 2015 are homogeneous (P<0.05) for each of the three cancers.

In our earlier report [18] we summarised the findings on aspirin and the incidence of metastatic spread in four studies, which, when pooled gave a relative risk in patients on aspirin of 0.77 (95% CI 0.86, 0.92; heterogeneity P<0.0005). Six reports in the studies published since March 2015 report metastatic spread, and the relationships with aspirin in these is summarised in Table 2 (HR 0.31 (95% CI 0.18, 0.51; Heterogeneity P = 0.89).

**Table 2:** Association between aspirin taking and metastatic spread.

Author	Cancer	Numbers ASA/Control	Estimates of reduction (95% CI)
Restivo et al. [26]	Rectum	37/204	HR 0.31 (0.18, 0.54)
Shiao et al. [36]	Breast	65/157	HR 0.34 (0.15, 0.81)
Osborn et al. [38]	Prostate	147/142	HR 0.23 (0.06, 0.91)
Rosenberg et al. [48]	Colorectal	49/191	OR 0.96 (0.65, 1.40)
Sanbury et al. [49]	Colorectal	75/34	OR 0.77 (0.40, 1.48)
Leitzmann et al. [50]	Prostate	16/33,076	RR 0.71 (0.31, 1.62)
**Pooled HR 0.31** (95% CI 0.18, 0.51) Heterogeneity P = 0.89; Egger’s test for bias 0.138			

We then proceeded to pool the results of all the available published reports, that is, both the 42 results in papers identified in our literature search to March 2015 [18] and the 29 in the recent search April 2015 to August 2017 described above. A flow diagram is given below in S1 Fig. Tests of significance of differences in the mean estimates given for the three main cancers in the two reviews, gave no evidence of heterogeneity (for all three: P>0.10). The review that follows is therefore based on 71 published reports, which together describe a total of over 120 thousand patients taking aspirin.

Assessment and grading of each of the reports, using the Newcastle-Ottawa grading scheme and including the papers which give evidence on metastatic spread, is given in S2 File.

### Colorectal cancer

Only one small *ad hoc* randomised trial of aspirin in 66 patients with colorectal cancer appears to have been reported. This showed an HR of 0.65 (95% CI 0.02, 18.06) for the mortality of patients randomised to aspirin.[12] A semi-randomised study of patients with another gastrointestinal cancer, oesophageal cancer, reported on 445 patients admitted to two wards, in only one of which aspirin was prescribed, showed an HR for aspirin of 0.83 (95% CI 0.68, 1.10). [15]

A total of 29 observational studies of aspirin and colorectal cancer have been reported and are listed in Table 1 and in our earlier report [18]. Twenty-seven give data on cause specific mortality and in 24 the measure of association with aspirin suggests benefit, 15 significantly (at P<0.05). In three studies [25,30,32] the measure of association exceeded 1.00 but in none of these three is the association significant (at P<0.05).

Further analyses are limited because the reports of effects by different authors use different indices: hazard ratios, risk ratios and odds ratios and these cannot be pooled together. However, twenty-one authors report the association with aspirin as hazard ratios, and these give a pooled HR 0.72 (95% CI 0.64–0.81; heterogeneity 0.0005) Eggers test is significant (P = 0.02) suggesting some publication bias. A forest plot and a funnel plot are shown in S3 File, and the removal of one outlying study [22] which appears to have excessive influence reduces the heterogeneity (P<0.001) and gives HR 0.75 (95% CI 0.68–0.83). We were unable to reduce heterogeneity further.

Evidence of a reduction in the occurrence of metastatic spread in colorectal cancer associated with aspirin comes from three studies, two of which report ORs [48,49] and together give 0.91 (95% CI 0.65, 1.26; heterogeneity P = 0.569). Another study [26] reported a reduction in rectal metastases in patients on aspirin as HR 0.31 (95% CI 0.18, 0.54).

Twenty-one of the studies of colorectal cancer also report all-cause mortality. In 19 the association with aspirin suggest a reduction with aspirin and in ten the reduction is significant. In two studies [27,31] the association with aspirin is consistent with an excess in all-cause deaths and in both the association is significant (at P<0.05). Eighteen studies of colorectal cancer report all-cause mortality as hazard ratios and pooling these gives HR 0.80 (95% CI 0.72, 0.89; heterogeneity P<0.0005; Eggers test for bias P = 0.002). The omission of three studies [22,23,27], selected on the basis of sensitivity analyses, reduced heterogeneity (P = 0.03) and gave HR 0.78 (95% CI 0.72, 0.85)

Overall therefore, the evidence on colorectal cancer is consistent that aspirin is associated with a reduction in colorectal mortality and a probable reduction in the incidence of metastatic spread. Egger’s test however suggests that there may be some publication bias in the available data for this cancer.

### Breast cancer

Our searches identified no study of aspirin randomised to patients with breast cancer. However, 14 observational studies listed in Table 1 and in our earlier report [18] have been reported, and in eleven the association suggests benefit, significant in six studies. Three of the studies report associations of 1.00 or greater with aspirin, but in none of these is the association significant.

Eight studies report the association with aspirin as a hazard ratio, and pooling these gives HR 0.69 (95% CI 0.53, 0.92; heterogeneity 0.0005 and Egger’s test for bias P = 0.14). The exclusion of one study [51] reduces heterogeneity (P = 0.80) and gives HR 0.80 (95% CI 0.66, 0.97) (see S3 File).

On the occurrence of metastatic spread in breast cancer, evidence of a reduction with aspirin is shown in three studies. Two [52,53] give a combined RR of 0.92 (95% CI 0.86. 0.99) and another study [36] gives HR 0.34 (0.15, 0.81).

Seven studies of breast cancer also reported all-cause mortality. In five the association with aspirin suggested benefit and in three the reduction in mortality associated with aspirin was significant. However, in two [36,54] the association indicated an excess in all-cause deaths in patients on aspirin, and in one of these the excess was significant at P<0.05. Six studies of breast cancer give a pooled estimate of all-cause mortality as HR 0.79 (95% CI 0.54, 1.16; heterogeneity p = 0.0005 and Egger's test for bias p = 0.74). The omission of three studies removes heterogeneity (p = 0.33) and gives HR for total mortality 0.97(95% CI 0.77, 1.21 see S3 File).

Overall therefore, there is evidence of benefit from aspirin taken by patients with breast cancer as an adjuvant treatment in terms of a reduction in breast cancer deaths and a significant reduction in the incidence of metastatic spread. Evidence of a reduction in all-cause mortality of these patients is suggestive, but not consistent across the studies.

### Prostate cancer

Our searches identified 16 observational studies, listed in Table 1 and in our earlier report. [18] Fifteen focused on prostate cancer mortality and in ten the index of association suggested a reduction associated with aspirin, significant in three. In five studies the measure of association with aspirin exceeded 1.00, and in one the excess in prostate mortality with aspirin is significant.

A pooled HR based on the thirteen studies that report the association with aspirin using this index is 0.87 (95% CI 0.73, 1.05; heterogeneity p<0.0005 and Egger’s test P = 0.13). The omission of one out-lying study [53] gives HR 0.84 (95% CI 0.77, 0.92; heterogeneity p = 0.17; see S3 File).

Evidence on metastatic spread in prostate cancer associated with aspirin, comes from four studies. Pooling three of there, gives RR 0.52 (0.39–0.68), and another study [38] gives HR 0.23 (0.06, 0.91).

Five studies on prostate cancer also reported all-cause mortality and three show a reduction associated with aspirin, significant in two. However, in two other studies [41,55] the association with aspirin exceeded 1.00 and was significant in both. Four studies report the association with aspirin as hazard ratios and together these give HR 0.84 (95% CI 0.54, 1.30; heterogeneity P<0.0005 and Egger’s test for bias P = 0.42). The omission of one outlier [55] removes heterogeneity and gives for all cause mortality in the prostate studies, HR 0.73 (95% CI 0.66, 0.81; see S3 File).

The evidence on prostate cancer is therefore fairly consistent for both prostate cancer mortality, and the hazard ratio for all cause mortality also suggests benefit. Correspondence with the author of the one report that is seriously inconsistent [55] led to no clue as to why it differs markedly from the other studies. The reduction in metastatic spread associated with aspirin is otherwise marked and consistent. In one report [42], it is suggested that some inconsistencies in studies of this cancer may be introduced by a selective up-take of PSA (prostate specific antigen) screening, leading to bias resulting from an early diagnosis of cancer in subjects taking aspirin.

### Other cancers

Our searches identified 15 reports on aspirin and other cancers (listed in Table 1 and in our earlier report. [18] No study showed significant evidence of detriment from aspirin specific mortality, or all-cause mortality. There is however suggestive evidence of benefit in 10 of the 15 studies, significant in six. In addition to the reports included in Table 1 we earlier reported evidence on ovarian cancer (HR 0.92 95% CI 0.81,1.06 [56], lung cancer (HR 0.84) [57], bladder (OR 0.75 95% CI 0.45, 1.24) [58], a mix of women’s cancers (HR 0.82; 95% CI 0.57, 1.18) [59] and lymphocytic leukaemia (HR 0.40; 95% CI 0.21, 0.79) [60]. There have also been two reports on head and neck cancer which differ markedly [47,61]. Associations between aspirin and metastatic spread are shown in Table 2, and there is also an earlier report on aspirin and endometrial cancer spread (HR 0.23; 05% CI 0.06, 0.91) [44].

While these single reports are a rather uncertain basis for clinical intervention, they very strongly give encouragement for the conduct of further studies, both observational and randomised.

### All cancers

Pooling the association of aspirin with all the cancers in our reports which have been reported as hazard ratios gives 74 (95% CI 0.66, 0.82) for cancer mortality and 0.81 (95% CI 0.73, 0.89) for all-cause mortality. Several published reports give similar estimates for total cancer mortality. A study based on 11,001 men taking aspirin and followed for 15 years, reported an overall reduction in all cancer deaths (HR 0.76; 95% CI 0.70, 0.82) [30]. In a report based on the US studies NHANES I and II, Ratnasinghe et al [32] give an RR of 0.98 (95% CI 0.84, 1.14) for cancer mortality in 14,838 subjects taking aspirin. Elsewhere, Algra & Rothwell give pooled estimates of reductions in total cancers in overviews of three previously randomised vascular trials of aspirin (OR 0.48; 95% CI 0.30, 0.75) [3], and these authors also give an overall estimate for the reduction in metastatic spread in long-term studies in which aspirin had originally been randomised: OR 0.69 (95% CI 0.57, 0.83).

Pooled estimates of the reductions in metastatic spread associated with aspirin in different cancers are also suggestive of benefit: in our earlier report [18] estimates reported in five studies give a pooled RR of 0.77 (95% CI 0.65, 0.92; heterogeneity P = 0.002) and three of the studies shown in Table 2 give a pooled HR of 0.31 (95% CI 0.20, 0.48; heterogeneity P = 0.89).

### Reports on bleeding

In our earlier review [18] we stated that four authors mentioned in their reports that no patient had experienced a major bleeding event, and in 21 answers to an email sent to the corresponding author of each report, none reported a major bleeding event. Again, the recent reports since March 2017 were scanned and all the corresponding authors were written to and asked about bleeding. The author of one report supplied data which showed that aspirin had not been associated with any significant excess in either serious bleeding (HR 1.11 95% CI 0.85, 1.44) or in fatal bleeding: 3% of the bleeds had been fatal in patients taking aspirin and 3.2% had been fatal in patients not taking aspirin [27]. Another author stated that within their cohort of 120 patients with liver cancer, six patients taking aspirin and seven not on aspirin had had a fatal gastrointestinal bleed [45]. A further author of a report with 491 patients [25] stated that a single bleeding event occurred in a patient on antiplatelet treatment, and three events occurred in patients not on antiplatelet treatment’. Three other authors stated than no major bleeding had occurred in the patients they had followed.

## Discussion

Ever since Gasic and colleagues reported in 1968–84 a series of pioneering studies on the role of platelets in the metastatic spread of cancer and a reduction in the incidence of metastases with aspirin [62–63] there has been an increasing interest in aspirin and cancer. Now, there is extensive experimental evidence on how platelets and the coagulation system protect tumour cells within the circulation from immune elimination, enable cancer cells to adhere to vascular endothelium and enhance the growth of the metastatic cells [64]. With this knowledge a reduction in metastatic spread by aspirin is a highly reasonable expectation.

The first evidence of an effect by aspirin on metastatic spread in human subjects came in one of the long-term follow-up studies by Rothwell and colleagues [11]. Aspirin was reported to be associated with significant reductions in metastases across a range of cancers, both in subjects with metastases at initial diagnosis and in the risk of later metastases in patients who had been free of metastases at diagnosis. In a later report based on long-term follow-up of participants in 51 vascular randomised trials, Rothwell et al commented on a reduction in short-term cancer mortality which they judged was too rapid to be attributable to ‘prevention’ and much more likely to be a ‘treatment’ effect [6].

Cochrane and others have stressed the importance of replication in science. This paper presents a replication of an earlier review [18] and then, because the estimates of effect of aspirin are comparable in the two reviews, we have based overall conclusions on meta-analyses of the pooled results from the two reviews. These results give extensive evidence consistent with reductions of about 15–25% in cancer mortality by aspirin. The evidence suggests however that there may be different levels of benefit in different cancers. Thus, there appears to be about a 25% reduction with aspirin in the mortality of colon cancer (HR 0.75; 95% CI 0.68–0.83), about 20% reduction in breast cancer mortality (HR 0.80; 95% CI 0.66, 0.97) and a probable 15% reduction in prostate cancer deaths (HR 0.86 (95% CI 0.78, 0.95). There is also evidence of a substantial reduction in the incidence of metastatic spread of these cancers, together with a reduction in all-cause mortality across all the cancers.

Almost all the evidence we present is, however, from observational studies within which the taking of aspirin is selective, leading to a number of important uncertainties. First, there is marked heterogeneity between the studies and our limited success in reducing this limits confidence in interpretation. There are many sources of possible differences between the series of patients in the various studies—differences in age and social factors, differences in other treatments and in general clinical management—and heterogeneity is probably inevitable, but at the same time it seems unlikely that such differences could generate the overall benefits we find to be associated with aspirin taking.

There are many sources of possible bias and in reviewing the present reports we were impressed by the frequency with which authors included evidence that the patients taking aspirin were older than patients not taking aspirin, and had a higher prevalence of co-morbidity, usually because of prevalent cardiovascular disease. Both these differences will operate against the detection of possible benefit being shown for aspirin.

In a commentary that details a number of possible sources of bias, Frouws and colleagues comment that ‘oncologists may withhold aspirin treatment in the most seriously ill patients because of the poor prognosis, leading to reverse causality’ [65]. We see no way to examine this particular bias, and yet, on the other hand, if physicians had withdrawn aspirin from patients who were seriously ill or showed marked deterioration this could have led to a rebound in vascular deaths [66–68], and the evidence presented for all-cause mortality makes it seem likely that if any such a process did occur, it must have been minor.

A recent paper by Rothwell et al [69] based on the long-term follow-up of five primary trials of aspirin and vascular disease, investigated possible interactions between age/body weight/dose of aspirin and the 20-year incidence of colon cancer. In brief, while low-dose aspirin (75–100 mg) was associated with a significant reduction in participants who weighed less than 70Kg, significance was lost in subjects weighing 70Kg and over. With reference to aspirin and the treatment of cancer, the report suggests ‘that low-dose aspirin might accelerate growth of some existing cancers at lower body size, particularly at older ages.’ A number of randomised trials of aspirin as an adjunct treatment of cancer are in progress [16, 70–73] and these will give opportunity to test any interactions of benefit with age, body weight and dose of aspirin. In the meantime, observational evidence is the main basis for decisions on the use of aspirin in cancer treatment.

Iatrogenic bleeding attributable to aspirin is clearly a most important issue. It is important however to consider not only the frequency but also the severity of bleeding, and to evaluate this in comparison with the likely benefits of aspirin taking, and in particular the reduction in all-cause mortality attributable to aspirin [74]. The evidence on bleeding summarised earlier in this report gives a measure of reassurance on bleeding and on fatal bleeding attributable to aspirin. Elsewhere, however, [75] we have reported a careful examination of the published evidence on fatal bleeding attributable to aspirin, showing than the proportion of fatal bleeds which occurred in subjects randomised to aspirin is lower that deaths due to spontaneous bleeding in subjects not on aspirin (RR 0.45; 95% CI 0.25, 0.80), and overall, in 52,583 subjects randomised to take aspirin, who together experienced 261 bleeding events, there was no significant increase in fatal bleeds in the subjects on aspirin, compared to spontaneous fatal bleeds in subjects randomised not to receive aspirin (RR 0.77; 95% CI 0.41, 1.43 P<0.91) [75]. A recent report describes 200,000 new users of low-dose aspirin, matched with a 1:1 cohort of non aspirin users [76]. During a follow-up of 5.4 years there was an excess in total gastrointestinal bleeds among the patients taking aspirin, but no excess in fatal bleeds (Rodríguez G, Lanas A, 2018. Personal communication. July, 19).

Furthermore, proton pump inhibitor drugs (PPIs) provide a high level of protection from intestinal bleeding whatever its aetiology [77,78], and formulae to assist in judging the risk of a gastrointestinal bleed in a subject are available [79,80].

There is also the issue of venous thromboembolism associated with cancer, and aspirin appears to be an effective prophylactic [81,82]. Patients with malignancy appear to be in a hypercoagulable state [83] with marked increases in both incidence and mortality of venous thromboembolism [84,85] and The American Society of Clinical Oncology has recommended that prophylactic anticoagulants be considered for all hospitalized cancer patients [86].

A plea for better and more complete information on aspirin and cancer has been made by representatives of the general public in a Citizens’ Jury held in 2006 [87] and the jurors added to their plea the phrase: ‘even before there is agreement between doctors’. A recent judgement by the UK Supreme Court went further and established that if a patient had not had opportunity to review all reasonable variant treatments and to express his/her views in a dialogue between doctor and patient, then the process of ‘informed consent’ could be called into question.[88] The court added to this judgement that if information is material, doctors should generally disclose it and should not wait for the patient to ask. Unfortunately however, knowledge of the likely benefit from aspirin is limited, and apprehension about the possible side effects and attitudes towards aspirin amongst primary care physicians is likely to impede acceptance of the growing evidence of benefit.[89]

## Implications for clinical practice and for research

A number of randomised trials are in progress. It will however be some years before these report and evidence will be limited to the more common cancers: colon [16,70–73], oesophageal [16], breast [16], prostate [16,73] and lung. One trial [16] will also give evidence on aspirin dose.

In the meantime, observational evidence is the main basis for decisions on the use of aspirin as an additional treatment of cancer. There is much favourable evidence on the three main cancers, but very little on the less common cancers, though what is available is, on the whole, encouraging. Because of the various uncertainties, the adequate informing of patients of the benefits and the harm is difficult, but it is important that shared decision making is not compromised by ‘intrusive external decisions’ [90].

Every possible effort should be made to encourage observational studies. More information is required on markers of likely benefit from aspirin such as the PIK3CA mutation, and on the optimal dose of aspirin for treatment, taking account of age, body weight smoking and possibly other personal factors.

Finally, valid evidence on serious and fatal bleeding attributable to aspirin, is urgently required. An increase in bleeding with age has been well documented, but so also has an increase in the benefits of aspirin and the balance between these outcomes needs to be evaluated in different groups of subjects and patients.

## Supporting information

S1 PRISMA Checklist (DOC)

S1 Table Aspirin treatment of cancer in observational studies reported 2015–2017. For details of studies published before 2015, see our earlier report [18].
(TIF)

S2 Table Association between aspirin taking and metastatic spread. (TIF)

S1 Fig Flow diagram. (TIF)

S1 File The search strategy to 31st August 2017. (DOCX)

S2 File Quality grading of each paper, with a Newcastle-Ottawa score. (DOCX)

S3 File Exploration of heterogeneity. (DOCX)
