# Supplementary figures and images for: Systematic review update of observational studies further supports aspirin role in cancer treatment: Time to share evidence and decision-making with patients?
Source: PLoS One. 2018 Sep 25;13(9):e0203957. doi: 10.1371/journal.pone.0203957 (PMC6155524; doi:10.1371/journal.pone.0203957)

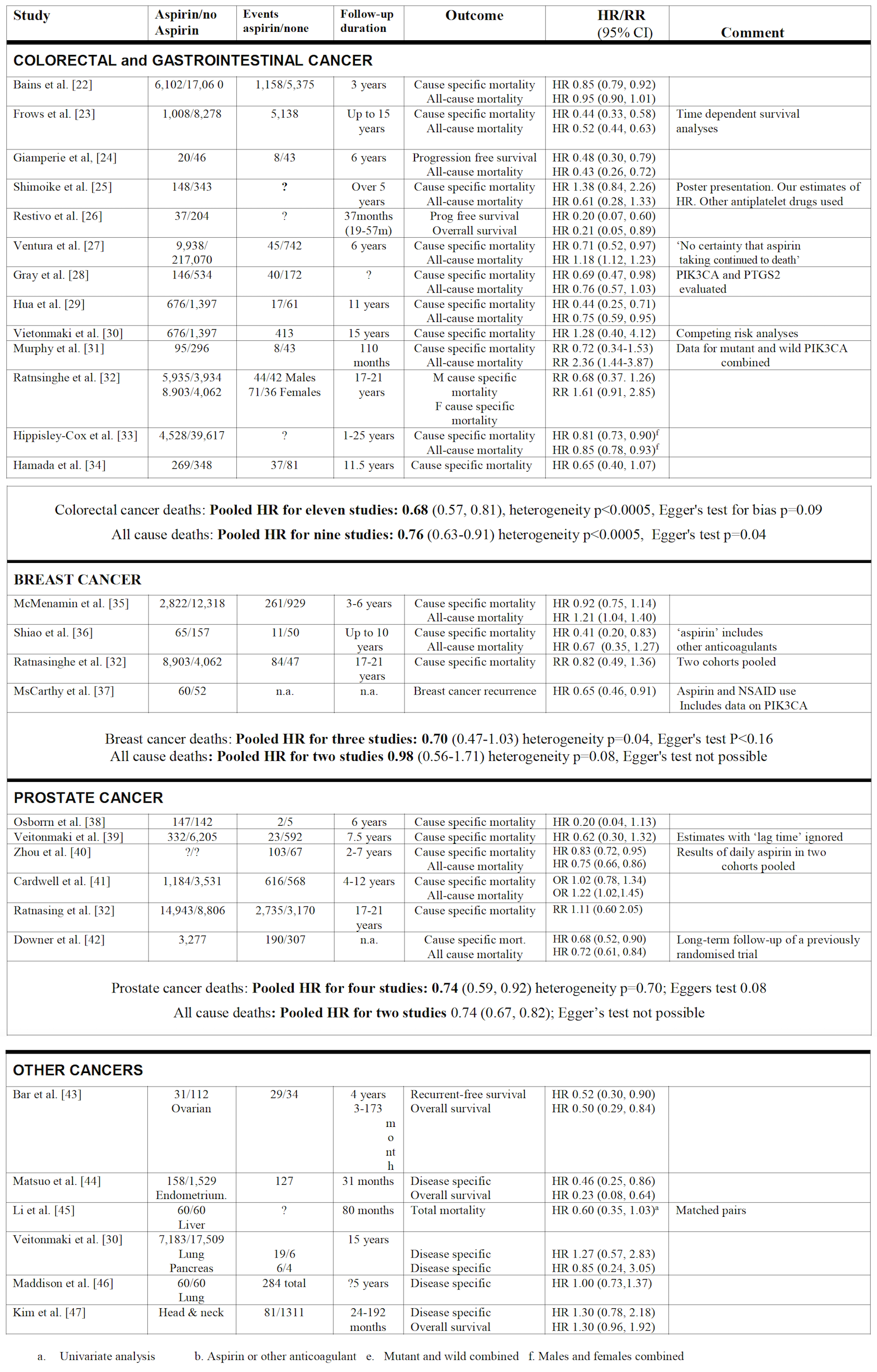

Supplement: S1 Table — For details of studies published before 2015, see our earlier report [18]. (TIF) [file pone.0203957.s002.tif]

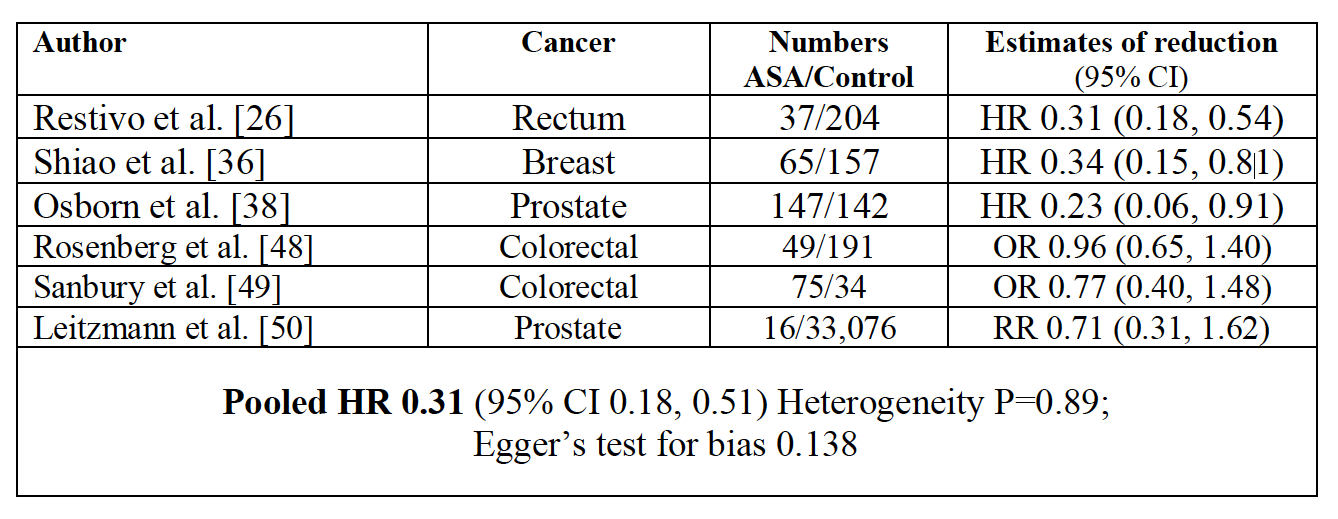

Supplement: S2 Table — (TIF) [file pone.0203957.s003.tif]

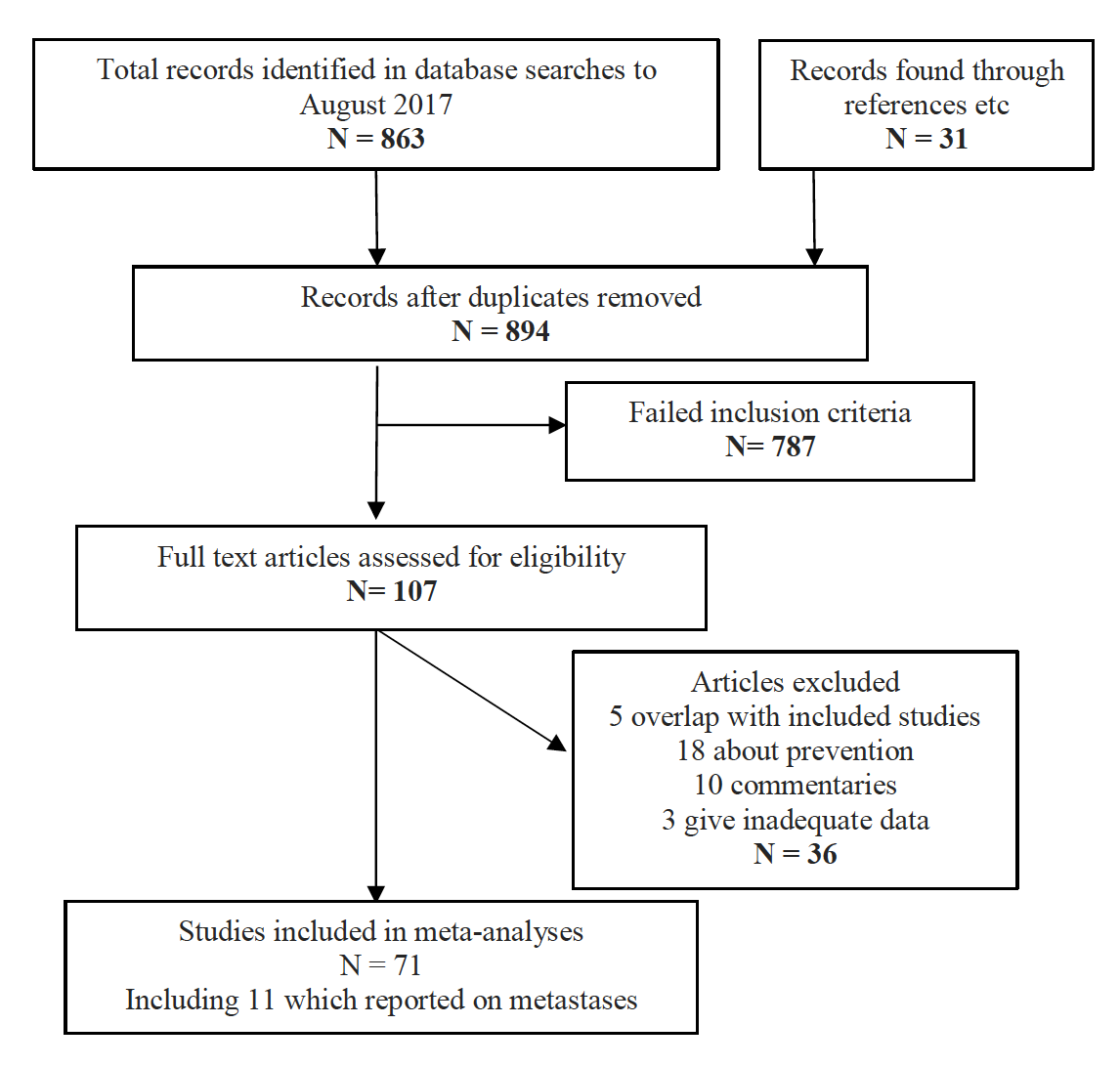

Supplement: S1 Fig — (TIF) [file pone.0203957.s004.tif]
