# Supplementary material for: Systematic review update of observational studies further supports aspirin role in cancer treatment: Time to share evidence and decision-making with patients?
Source: PLoS One. 2018 Sep 25;13(9):e0203957. doi: 10.1371/journal.pone.0203957 (PMC6155524; doi:10.1371/journal.pone.0203957)
Supplement: S1 File — (DOCX) [file pone.0203957.s005.docx]

**23^rd^ October 2017**

**SUPPLEMENT**

**to**

**PONE-D-18-08743 Systematic review update of observational studies**

**further supports aspirin role in cancer treatment:**

**time to share evidence and decision-making with patients?**

Elwood PC, Pickering JE, Morgan G, Galante J, Weightman AL, Morris D,

Longley M, Mason M, Adams R, Dolwani S, Chia JWK, Lanas A

S1. The search strategy

S2. Summary of each paper with an assessment and a quality score, according to the Newcastle-Ottawa scheme.

S3. Further analyses:

Forest plots, Funnel plots and Sensitivity analyses

conducted in attempts to reduce heterogeneity.

3a. Colorectal cancer mortality

3b. All cause mortality in papers on colorectal cancer

3c. Breast cancer mortality

3d. All cause mortality in papers on breast cancer

3e. Prostate cancer mortality

3f. All cause mortality in papers on prostate cancer

**PONE-D-18-08743 Systematic review update of observational studies**

**further supports aspirin role in cancer treatment:**

**time to share evidence and decision-making with patients?**

**S1. The search strategy to 31^st^ August 2017.**

Systematic searches were conducted in Medline and Embase, using the key words: “aspirin”, “acetylsalicylic acid”, “cancer” “tumour”, “neoplasm”, “mortality”, “death”, “adverse effect”, “bleed”. The searches were limited to human studies in peer-reviewed journals. Reference lists of the relevant studies identified were also searched. Studies were selected by two authors (PE and GM) if (a) the studied population comprised patients diagnosed with cancer; (b) aspirin was taken regularly after cancer diagnosis; (c) they were randomised trials, case-control studies or cohort studies. Cancer specific, all-cause mortality and incidence of metastases and adverse effects were noted.

**Search strategy developed using the following search filters for study design.**

- Observational studies: SIGN filter  (<http://www.sign.ac.uk/methodology/filters.html#obs>)
- Randomised controlled trials : Cochrane highly sensitive search filter (<http://handbook.cochrane.org/chapter_6/box_6_4_c_cochrane_hsss_2008_sensmax_ovid.htm>)

**MEDLINE and Medline in Process**

1. randomized controlled trial.pt.
2. controlled clinical trial.pt.
3. randomized.ab.
4. placebo.ab.
5. drug therapy.fs.
6. randomly.ab.
7. trial.ab.
8. groups.ab.
9. or/1-8
10. Epidemiologic studies/
11. exp case control studies/
12. exp cohort studies/
13. Case [control.tw](http://control.tw).
14. (cohort adj (study or studies)).tw.
15. Cohort analy*.tw.
16. (Follow up adj (study or studies)).tw.
17. (observational adj (study or studies)).tw.
18. Longitudinal.tw.
19. Retrospective.tw.
20. Cross [sectional.tw](http://sectional.tw).
21. Cross-sectional studies/
22. or/10-21
23. 9 or 22
24. exp animals/ not humans.sh.
25. 23 not 24
26. Exp neoplasms/
27. (cancer* or malign* or tumour* or tumor*).tw
28. 26 or 27
29. Aspirin/
30. (aspirin* or "acetylsalicylic acid").tw
31. 29 or 30
32. 25 and 28 and 31
33. Limit 32 to year=2017
    **EMBASE**
34. Random*.tw
35. Clinical trial*.mp
36. Exp health care quality/
37. placebo.ab.
38. or/1-4
39. clinical study/
40. case control study/
41. family study/
42. longitudinal study/
43. retrospective study/
44. prospective study/
45. Cohort analysis/
46. (Cohort adj (study or studies)).mp.
47. (Case control adj (study or studies)).tw.
48. (follow up adj (study or studies)).tw.
49. (observational adj (study or studies)).tw.
50. (epidemiologic* adj (study or studies)).tw.
51. (cross sectional adj (study or studies)).tw.
52. or/6-18
53. 5 or 19
54. exp animal/ not human.sh.
55. 20 not 21
56. Exp neoplasms/
57. (cancer* or malign* or tumour* or tumor*).tw
58. 23 or 24
59. Aspirin/
60. (aspirin* or "acetylsalicylic acid").tw
61. 26 or 27
62. 22 and 25 and 28
63. Limit 29 to “year=2017”
