# Supplementary material for: Systematic review update of observational studies further supports aspirin role in cancer treatment: Time to share evidence and decision-making with patients?
Source: PLoS One. 2018 Sep 25;13(9):e0203957. doi: 10.1371/journal.pone.0203957 (PMC6155524; doi:10.1371/journal.pone.0203957)
Supplement: S2 File — (DOCX) [file pone.0203957.s006.docx]

**PONE-D-18-08743 Systematic review update of observational studies**

**further supports aspirin role in cancer treatment:**

**time to share evidence and decision-making with patients?**

**S2. Quality grading of each paper**, with a Newcastle-Ottawa score

Cohort studies

[Newcastle Ottawa Assessment Scale](http://www.ohri.ca/programs/clinical_epidemiology/nosgen.pdf)

| Study | Exposed cohort representative | Non exposed selection | Exposure ascertainment | Outcome not at start?^$^ | Controls for age | Controls for other factors | Outcome assessment | Follow-up long enough ≥3 years | Follow-up adequate | Score | General comments |
| --- | --- | --- | --- | --- | --- | --- | --- | --- | --- | --- | --- |
| Assayag 2014 | * | * | * | * | * | * | * | * | * | 9 |  |
| Assayag 2015 | * | * | * | * | * | * | * | * | * | 9 |  |
| Bains 2015 | * | * | * | * | - | * | * | - | * | 7 | Conference proceeding only. Multivariate analysis but unclear if controlled for age. Median follow up only 2.2 years |
| Bains 2016 | * | * | * | * | * | * | * | * | * | 9 | Linked to Bains 2015. Post-diagnosis aspirin use only. |
| Bar 2016 | - | * | * | * | * | * | * | * | * | 8 | Smallish and selected group from single centre (258) that may not be representative. |
| Barron 2014 | - | * | * | * | * | * | * | * | * | 8 | Only participants entitled to free prescriptions were included thus ~ 1/3 total population |
| Barron 2015 | - | * | * | * | - | - | - | - | - | 3 | Abstract only. Data for 764/4540 member of registry (16.8%). May not be representative. No information on whether controlled for confounders, time or adequacy of follow-up. |
| Bastiaannet 2012 | * | * | * | * | * | * | * | * | * | 9 |  |
| Blair 2007 | * | * | - | * | * | * | * | * | * | 8 | NSAID use assessed by written self report only – not med record or interview |
| Bowers 2014 | - | * | * | - | * | * | * | * | * | 7 | Retrospective – medical record review. Single centre only N=440; may not be representative. Recurrence may have been there at start (even if not clinically apparent). Median follow up not provided but 14 year time frame and time to recurrence data suggest >>3 years. |
| Bradley 2016 | * | - | - | * | * | * | * | * | * | 7 | Participants drawn from RCT. Aspirin use by self report only and dosage unclear. Aspirin users had greater comorbidity and more likely to be current smokers and ibuprofen users. Authors say asprin users were older but not backed up in Table 1 which only gives mean age at diagnosis. |
| Caon 2014 | * | * | - | * | * | * | * | * | * | 8 | Retrospective – chart review. ASA medication coded from initial consultation only. No duration data. |
| Chae 2013 | - | * | - | * | - | - | * | - | - | 3 | Abstact only. Single centre targeted-therapy trial population (N=296); may not be representative. Not clear how aspirin use was measured. No indication of adjustment for age (adj for Royal Marsden Score only) and aspirin users were older (mean difference 8.6 years, p<0.001). Follow up time and adequacy unstated. |
| Chae 2014 | - | * | - | * | - | * | - | * | * | 5 | Only a small number taking aspirin – 21/280. No description of exposure or outcome assessments |
| Chan 2009 | * | * | - | * | * | * | * | * | * | 8 | Aspirin use assessed by written self report only – but a validated assessment |
| Choe 2012 | * | * | - | * | - | * | * | * | * | 7 | Aspirin use assessed by written self report only. No apparent control for age even though baseline differences noted. |
| Coghill 2011 | * | * | * | * | * | * | * | * | * | 9 |  |
| Cronin-Fenton 2015 | * | * | * | - | - | * | - | * | - | 5 | Conference abstract only. Unclear how recurrence was measured and confirmed that outcome not there at start of study. Adjusted for confounders but unclear if age was one of them. |
| Daugherty 2013 | * | * | - | * | * | * | * | * | * | 8 | Aspirin use assessed by written self report only |
| Dhillon 2012 | * | * | - | * | * | * | * | * | * | 8 | Aspirin use assessed by written self report only. Explored mortality and metastases (latter may have been present at start). |
| Din 2010 | * | - | * | * | * | * | - | * | - | 6 | Only ~45% of incident cases included and no description of excluded vs included differences; exposure ascertained by self report; response rates differ for cases (48%) and controls (61%) |
| Domingo 2013 | * | * | * | * | * | * | * | * | * | 9 | Trial population cohort (refecoxib) but N=896 and multicentre |
| Downer 2017 | - | * | * | * | * | * | * | * | * | 8 | Healthy male physicians only. May not be representative – likely higher social class. Aspirin use randomised as part of (original) trial and then confirmed by annual self report. NB high dose aspirin 325mg |
| Flahavan 2014 | - | * | * | * | * | * | * | * | * | 8 | Only participants entitled to free prescriptions were included – approx 1/3 of the total population. May not be representative. |
| Fontaine 2010 | * | * | * | * | * | * | * | * | * | 9 | Unclear how aspirin exposure was ascertained “...were recorded as regularly taking” but medical record; thus scored * |
| Fraser 2014 | * | * | * | * | * | * | * | * | * | 9 |  |
| Frouws 2017 [51] | * | * | * | * | * | * | * | * | * | 9 | Colon câncer PLOSone |
| Frouws 2017 [52] | * | * | * | * | * | * | * | * | * | 9 | Oseophageal câncer BJC. Linked to van Staalduinen 2016? |
| Frouws 2017 [NewFrouws1] | * | * | * | * | * | * | * | * | * | 9 | Colorectal câncer EJC. |
| Fuchs 2005 | * | * | - | * | * | * | - | - | - | 5 | Abstract only. Trial population cohort (adjuvant chemotherapy) but N=830 and multi-centre. Aspirin use assessed by written self report only. Median follow up only 2.4 years. Assessment of outcome unclear. Drop outs unclear. |
| Giampieri 2016 | - | * | - | * | * | * | * | - | * | 6 | Tiny study and highly selected group. N=66. Unclear how aspirin use was ascertained. Short follow up (advanced metastatic colon cancer). |
| Goh 2014 | * | * | * | * | * | * | * | * | * | 9 |  |
| Gray 2017 | * | * | * | * | * | * | * | * | * | 9 |  |
| Grytli 2014 | * | * | * | * | * | * | * | * | * | 9 |  |
| Hamada 2017 | - | * | - | * | ? | * | * | * | * | 7 | Health professionals only with specific tumour tissue and CD274 expression. N=617. Not representative of all rectal/colon cancer. Aspirin use assessed by written self report only. |
| Hippisley-Cox 2017 | * | * | * | * | * | * | * | * | * | 9 |  |
| Holmes 2010 | - | * | - | * | * | * | * | * | * | 7 | Registered nurses only. May not be representative. Aspirin use assessed by written self report only. |
| Hua 2017 | * | * | * | * | * | * | * | * | * | 9 |  |
| Jacobs 2014ª *Cancer Biol Ther* | - | * | - | * | * | * | * | * | * | 7 | Small selected group (n=74) may not be representative. Unclear how exposure was ascertained ‘*patients reported that....*’. Age of control group significantly younger than treatment group and some hormone differences but controlled for in analysis |
| Jacobs 2014b *J. Clin Oncol* | * | * | - | * | * | * | * | * | * | 8 | Aspirin use assessed by written self report only |
| Kim 2017 | - | * | * | * | * | * | * | * | * | 8 | Single centre study (N=1391) and only 81 aspirin users. May not be representative |
| Kothari 2014 | * | * | * | * | * | * | * | * | * | 9 | Aspirin users were significantly older and had higher proportion of left sided cancers but controlled for in analysis |
| Kwan 2007 | - | * | - | * | * | * | * | * | * | 7 | Only 46% agreed to participate in cohort – may not be representative. Aspirin use assessed by written self report only |
| Li 2016 [87] | * | * | - | * | * | * | * | * | * | 8 | Breast câncer. Conference abstract only. Single institution only though large population group (1000). Unclear how aspirin use was ascertained. |
| Li 2016 [83] | - | * | * | * | * | * | * | * | * | 8 | Hepatocellular câncer. Tiny single centre specific sample (aspirin plus chemoembolization; N=120) with matched no aspirin group. |
| Liao 2012 | - | * | * | * | * | * | * | * | - | 7 | Nurses and health professionals only. May not be representative. Aspirin use assessed by written self report only. Unclear whether follow-up was adequate. Just said that used data for 1097 with tumour, aspirin and survival information. |
| Ljung 2014 | * | * | * | * | * | * | * | - | * | 8 | Retrospective cohort. Younger women excluded (low aspirin use) but 80% population included so regarded as representative. Median/mean follow-up period not reported but described as short and whole study only 2006-2011 |
| Macfarlane 2015 | * | * | * | * | * | * | - | * | - | 7 | Unclear if follow up was adequate (see PCCIU web site – practices were withdrawing during study period) or how outcome was confirmed. |
| Maddison 2017 | - | * | * | * | * | * | * | - | * | 7 | Smallish study (313) and nuclear if truly representative (recruitment over an 8 year period from hospitals in Trent). Median follow up 9.75 months. |
| Matsuo 2016 | - | * | - | * | * | * | * | - | * | 6 | Very Small number exposed to low dose ASA (158) so may not be representative. Exposure ascertainment not clear. ‘*Medication history was verified through multiple sources’.* Median follow up 31.5 months |
| McCarthy 2017 | - | * | - | * | * | * | - | - | * | 5 | Conference proceeding only. Single centre study only (N=212) of HR+/HER2- breast câncer patients. Not representative. Exposure ascertainment nuclear. No information on mortality as outcome. No follow up time provided. |
| McCowan 2013 | * | * | * | * | * | * | * | * | * | 9 |  |
| McMenamin 2017 | * | * | * | * | * | * | * | * | * | 9 |  |
| Murphy 2016 | - | * | * | * | * | * | - | * | * | 7 | Smallish numbers (488) and difficult to confirm if ACCORD database is representative since just says ‘participating hospitals’ on web site. Outcome assessment not clear. ‘Survival data available via ACCORD’ |
| Ng 2015 | - | * | - | * | * | * | * | * | - | 7 | Cohort from an RCT of adjuvent chemotherapy. Self report and only 75/799 responded to aspirin question. |
| Osborno 2016 | - | * | - | * | * | * | * | * | * | 7 | Smallish and selected group (289) that may not be representative. Ascertainment of aspirin use not clear – defined as ‘*being used at start of radiation therapy or on subsequent followup’* |
| Pastore 2015 | * | * | * | - | * | * | * | * | * | 8 | Potential for outcome (tumour recurrence) to be there at start? |
| Ratnasinghe 2004 | * | * | * | * | * | * | * | * | * | 9 |  |
| Reimers 2012 | * | * | * | * | * | * | * | * | * | 9 |  |
| Reimers 2014 | * | * | * | * | * | * | * | * | * | 9 |  |
| Restivo 2015 | - | * | * | * | * | * | * | * | * | 8 | Single centre small study (241) so may not be representative. |
| Shiao 2016 | - | * | - | * | * | * | * | * | * | 7 | Single centre smallish study (222) so may not be representative. Ascertainment of aspirin use not clear – counted as yes if patients had ‘*listed aspirin on the medication list at start of treatment or anytime during followup’* |
| Shimoike 2014 | - | * | - | * | - | * | * | * | * | 6 | Conference proceeding only. Single institution only and smallish sample (491). Exposure ascertainment not stated. Unclear if age was controlled for. |
| Una 2017 | * | * | * | * | * | * | * | * | * | 9 |  |
| van Staalduinen 2016 | * | * | * | * | * | * | * | - | * | 8 | Linked to Frouws 2017? Median follow up for deceased patients only 0.55y. 3.3 y for survivors |
| Veitonmaki 2015 [155 & 155ª] | * | * | * | * | * | * | * | * | * | 9 | Cohort study building on RCT of prostate screening. Two papers from the same study? |
| Veitonmaki 2016 | * | * | * | * | * | * | * | * | * | 9 | Exposure was assessed via a drug purchase database (thus *) but also a questionnaire for over the counter use. The later would not receive a *. |
| Ventura 2016 | * | * | - | * | * | * | * | * | * | 8 | Aspirin exposure ascertainment only covered the first 12 month period of 2007 though patients followed up to Dec 2013. |
| Zanders 2015 | * | * | * | * | * | * | * | * | * | 9 | Although these are patients with diabetes, this is the same cohort as the Reimers 2014 study? Inclusion of results for both in a single meta-analysis – risk of double counting. |
| Zhou 2017 | - | * | - | * | * | * | * | * | - | 6 | Population may not be representative. Only 59% of subjects completed the risk factor questionnaire and only 66% of this number completed the follow-up questionnaire Aspirin use assessed by written self report only. |

**Case control studies**

[Newcastle Ottawa Assessment Scale](http://www.ohri.ca/programs/clinical_epidemiology/nosgen.pdf)

| Study | Case definition adequate? | Cases representative | Control selection | Definition of controls | Controls for age | Controls for other factors | Ascertainment of exposure | Same for cases/controls | Non-response rate | Score | Potential confounders |
| --- | --- | --- | --- | --- | --- | --- | --- | --- | --- | --- | --- |
| Cardwell 2014 | * | * | * | * | * | * | * | * | * | 9 |  |
| Din 2010 | * | - | * | * | * | * | - | * | - | 6 | Only ~45% of incident cases included and no description of excluded vs included differences; exposure ascertained by self report; response rates differ for cases (48%) and controls (61%) |
| Holmes 2014 | * | * | * | * | * | * | * | * | * | 9 | Authors found a modest co-morbidity and education effect. |
| Murray 2014 | * | * | * | * | * | * | * | * | * | 9 |  |
| Nagle 2015 | - | * | * | - | * | * | - | * | * | 6 | Case = aspirin use? Self report. Checked AOCS web site and 93% follow up. Aspirin use covered the period preceeding diagnosis so relevance? Discuss with Peter. |
| Rosenberg 1998 | * | * | * | * | * | * | * | * | * | 9 | Probably representative but only ca 65% of eligible cases and eligible controls responded |
| Sansbury 2005 | * | * | * | - | * | * | * | * | - | 7 | Assumed representative (N=1691) though no data provided on non-response rates. No information given on câncer history of controls. |

**Other studies**

| Study | Study design | Appraisal form used | Notes |
| --- | --- | --- | --- |
| Creagan 1991 | Controlled clinical trial? | [SURE RCT & other experimental studies appraisal form](http://www.cardiff.ac.uk/insrv/libraries/sure/doc/SURE_RCTs%20and%20other%20experimental%20studies_Checklist_2015%20update.pdf) | Assignment via Pocock-Simon stratification (to allocate to groups by balanced prognostic factors) and not randomisation (Controversial: Pond BJCancer 2011; 104(11): 1711-1715). No ethical approval reported. Patients and investigators not blinded – argued not needed since hard outcomes of progression/survival. No trial protocol. No power calculation. Treat as CCT? |
| Lebeau 1992 | RCT | [SURE RCT & other experimental studies appraisal form](http://www.cardiff.ac.uk/insrv/libraries/sure/doc/SURE_RCTs%20and%20other%20experimental%20studies_Checklist_2015%20update.pdf) | Control group not blinded (no placebo). No ethical approval reported. No trial protocol. No baseline characteristics provided. P values but no confidence intervals to describe precision of effect. No information on conflicts of interest. |
| Lipton 1982 | Controlled clinical trial? | [SURE RCT & other experimental studies appraisal form](http://www.cardiff.ac.uk/insrv/libraries/sure/doc/SURE_RCTs%20and%20other%20experimental%20studies_Checklist_2015%20update.pdf) | Method of randomisation not stated. Baseline differences. No power calculation and small numbers (66). Data analysis not well described and no precision of effect |
| Liu 209 | Controlled clinical trial | [SURE RCT & other experimental studies appraisal form](http://www.cardiff.ac.uk/insrv/libraries/sure/doc/SURE_RCTs%20and%20other%20experimental%20studies_Checklist_2015%20update.pdf) | Allocation to study group by ward and not randomised. No trial protocol. No power calculation but a large study (N=1598). No confirmation of compliance with aspirin. 93% follow-up and lack of response treated as having died. No conflict of interest reporting. |
