# Supplementary material for: Systematic review update of observational studies further supports aspirin role in cancer treatment: Time to share evidence and decision-making with patients?
Source: PLoS One. 2018 Sep 25;13(9):e0203957. doi: 10.1371/journal.pone.0203957 (PMC6155524; doi:10.1371/journal.pone.0203957)
Supplement: S3 File — (DOCX) [file pone.0203957.s007.docx]

**PONE-D-18-08743 Systematic review update of observational studies**

**further supports aspirin role in cancer treatment:**

**time to share evidence and decision-making with patients?**

**S3. Exploration of Heterogeneity**

**3a. Colorectal cancer**  cause specific mortality (21 studies)

HR 0.72 (0.64-0.80); Heterogeneity p<0.0005; Eggers test for bias: p=0.022

**Systematic review update of observational studies**

**further supports aspirin role in cancer treatment:**

**time to share evidence and decision-making with patients?**

**3a. Colorectal cancer**  cause specific mortality (21 studies)

3a. **Colorectal cancer**  cause specific mortality (21 studies)

Sensitivity Analysis:

Removing Frows^22^ because of possible excessive influence reduced heterogeneity to p=0.001 **HR 0.75 (0.68-0.83)**

We were unable to reduce heterogeneity further for colorectal cancer.

**3b. All cause mortality in papers on colorectal cancer** (18 studies)

HR 0.80 (0.72-0.89) Heterogeneity<0.0005 Egger's test for bias p=0.002

**3b. All cause mortality in papers on colorectal cancer** (18 studies)

Egger's test for bias p=0.002

**3b. All cause mortality in papers on colorectal cancer** (18 studies)

Heterogeneity reduced to p=0.03 by excluding Frows^22^, Zanders^63^, Bains^21^ and Ventura^26^ giving **HR 0.78 (0.72-0.85).**

3c. **Breast Cancer** cause specific (8 studies)

HR = 0.69 (0.52-0.93) Heterogeneity p< 0.0005

Egger's test for bias p=0.144

3c. **Breast Cancer** cause specific (8 studies)

3c. **Breast Cancer** cause specific (8 studies)

Egger's test for bias p=0.202

Sensitivity Analysis:

Heterogeneity reduced to p=0.049 by excluding Frazer^68^

giving **HR 0.80 (0.66-0.97).**

3d. **All cause mortality** in papers on breast cancer (6 studies)

HR 0.79 (0.54-1.16) heterogeneity p<0.0005 Egger's test for bias p=0.738

3d. **All cause mortality** in papers on breast cancer (6 studies)

Egger's test for bias p=0.738

3d. **All cause mortality** in papers on breast cancer (6 studies)

Sensitivity analysis

Heterogeneity reduced by excluding Frazer^68^, McMenamin^34^ and Blair^65^ **HR 0.97 (0.77-1.21)** Heterogeneity p=0.334

3e. **Prostate Cancer** cause specific mortality (13 studies)

HR= 0.87 (0.73-1.05) Heterogeneity p<0.0005; Egger’s test P+0.132

3e. **Prostate Cancer** cause specific mortality (13 studies)

3e. **Prostate Cancer** cause specific mortality (13 studies)

Egger's test for bias p=0.178

A non-significant level of heterogeneity achieved by excluding Assayag^75^ giving **HR= 0.84 (0.77-0.92)** Heterogeneity p=0.230

3F. Prostate cancer, all cause mortality (4 studies)

HR 0.84 (0.54-1.30) Heterogeneity p<0.0005

Egger's test for bias p=0.424

3f. **All cause mortality** in papers on prostate cancer (4 studies)

3f. **All cause mortality** in papers on prostate cancer (4 studies)

Sensitivity analysis

Heterogeneity again reduced by excluding Assayag^75^ giving HR 0.73 (0.66-0.81) Heterogeneity p=0.597
